# Identification of genes and pathways leading to metastasis and poor prognosis in melanoma

**DOI:** 10.18632/aging.203554

**Published:** 2021-09-28

**Authors:** Xin Zhang, Wandong Wang, Yun Wang, Guan Jiang

**Affiliations:** 1 Department of Dermatology, The Affiliated Hospital of Xuzhou Medical University, Xuzhou, China; 2 Xuzhou Medical University, Xuzhou, China

**Keywords:** melanoma, bioinformatics, signaling pathway, survival analysis, regulatory analysis

## Abstract

Melanoma causes the highest mortality rate among all skin cancers. However, the underlying molecular mechanisms leading to metastasis and poor prognosis in melanoma have not been fully elucidated. In this study, the differentially expressed genes (DEGs) related to metastasis in melanoma were screened out. The results of gene annotation was combined with The Cancer Genome Atlas (TCGA) database. The microRNA (miRNA) network that regulates key genes and their correlation with *BRAF^V600E^* was preliminarily analyzed. Cell and molecular biology experiments were conducted to verify the results of bioinformatics analysis. Results showed that the PI3K-Akt signaling pathway contained the key genes *CDK2*, *CDK4*, *KIT*, and *Von Willebrand factor*. Survival analysis showed that high expression of the four key genes significantly reduced the survival rate of patients with melanoma. Correlation analysis showed that *BRAF^V600E^* may regulate the expression of the four key genes, and a total of 240 miRNAs may regulate this expression. Experiments showed that the inactivation of key genes inhibits the proliferation, migration, and invasion of melanoma. In conclusion, the PI3K-Akt signaling pathway and the four key genes promoted the proliferation, migration, and invasion of melanoma, and related to poor prognosis of patients with melanoma.

## INTRODUCTION

Melanoma is a type of skin tumor caused by genetic mutations in melanocytes. Although melanoma accounts for a small proportion of all skin cancers, it causes the highest number of deaths among all skin cancers, close to 80% [1, 2]. When advanced melanoma is diagnosed, up to one-third of patients already have multiple lesions. Melanoma progresses rapidly, and metastatic lesions usually involve the brain and internal organs. Although a subset of patients who received targeted therapy or immune checkpoint inhibitor could have ongoing long-term tumor control, when melanoma progresses to stage IV, the patient’s 10-year survival rate is only 10%–15%. Thus, early treatment is preferred for patients with melanoma [3, 4]. Therefore, the mechanism leading to the initiation, progression, and invasion of melanoma has aroused the interest of researchers worldwide [5]. Early diagnosis and treatment could effectively prevent the metastasis and further progress of melanoma, and this prevention is the key to treating it and improving its prognosis.

Among the gene mutations in melanoma cells, the most common is *BRAF* mutation (accounting for 60% of all melanomas), which could lead to disordered cell signaling pathways and uninhibited proliferation [6]. V600E mutation is the most common mutation in *BRAF*, accounting for approximately 74%–86% [7]. The first drug to inhibit *BRAF* ^V600E^ was vemurafenib (PLX4032) [8, 9]. Another inhibitor that also inhibits the *BRAF^V600E^* gene is dabrafenib (GSK2118436), which has similar effects to vemurafenib [10]. The effect of this inhibitor is effective at first, but drug resistance often appears within a year, and the therapeutic effect gradually declines [11, 12]. Drug resistance reduces the efficacy of targeted therapy for metastatic melanoma. MEK inhibitors could enhance the inhibitory effect of the RAS/RAF/MEK/ERK mitogen-activated protein kinase (MAPK) pathway in *BRAF* mutant cells. By combining BRAF with MEK inhibitors, the resistance could be temporarily resolved [13]. This combination therapy has become the most effective treatment for patients with *BRAF^V600E^* mutant melanoma [14]. However, almost all patients still develop resistance within a few years [15, 16]. Therefore, identifying the reasons leading to the occurrence and development of melanoma, finding new therapeutic targets for melanoma, and providing more treatment options to combat this devastating disease have become an important research direction for scientists.

In recent years, microarray and bioinformatic technology have played an important role in analyzing and revealing the mechanism of the occurrence and development of various diseases [17]. Researchers used bioinformatics to analyze the results of sequencing, study the gene expression in samples, and make important contributions to clarifying the diagnosis of the disease, thereby leading to development prevention and prognostic improvement. The metastasis of melanoma involves various genes and signaling pathways closely related to the prognosis of patients. The use of bioinformatic methods to effectively identify key genes and pathways could help further clarify the molecular mechanism of the metastasis of melanoma and provide potential therapeutic targets.

In this study, melanoma samples in the Gene Expression Omnibus (GEO) and The Cancer Genome Atlas (TCGA) databases were analyzed to screen out differentially expressed genes (DEGs) associated with the metastasis of melanoma. Through a series of bioinformatic analyses and experiments, the key genes and pathways that may lead to this metastasis were found. Survival analysis revealed the effect of key genes on melanoma prognosis. In addition, some molecular regulatory mechanisms were found to affect the expression levels of these key genes. Therefore, this study may provide a new method for revealing the potential mechanism of the metastasis of melanoma and new therapeutic targets.

## RESULTS

### DEGs in the two groups of samples

**Figure 1:**
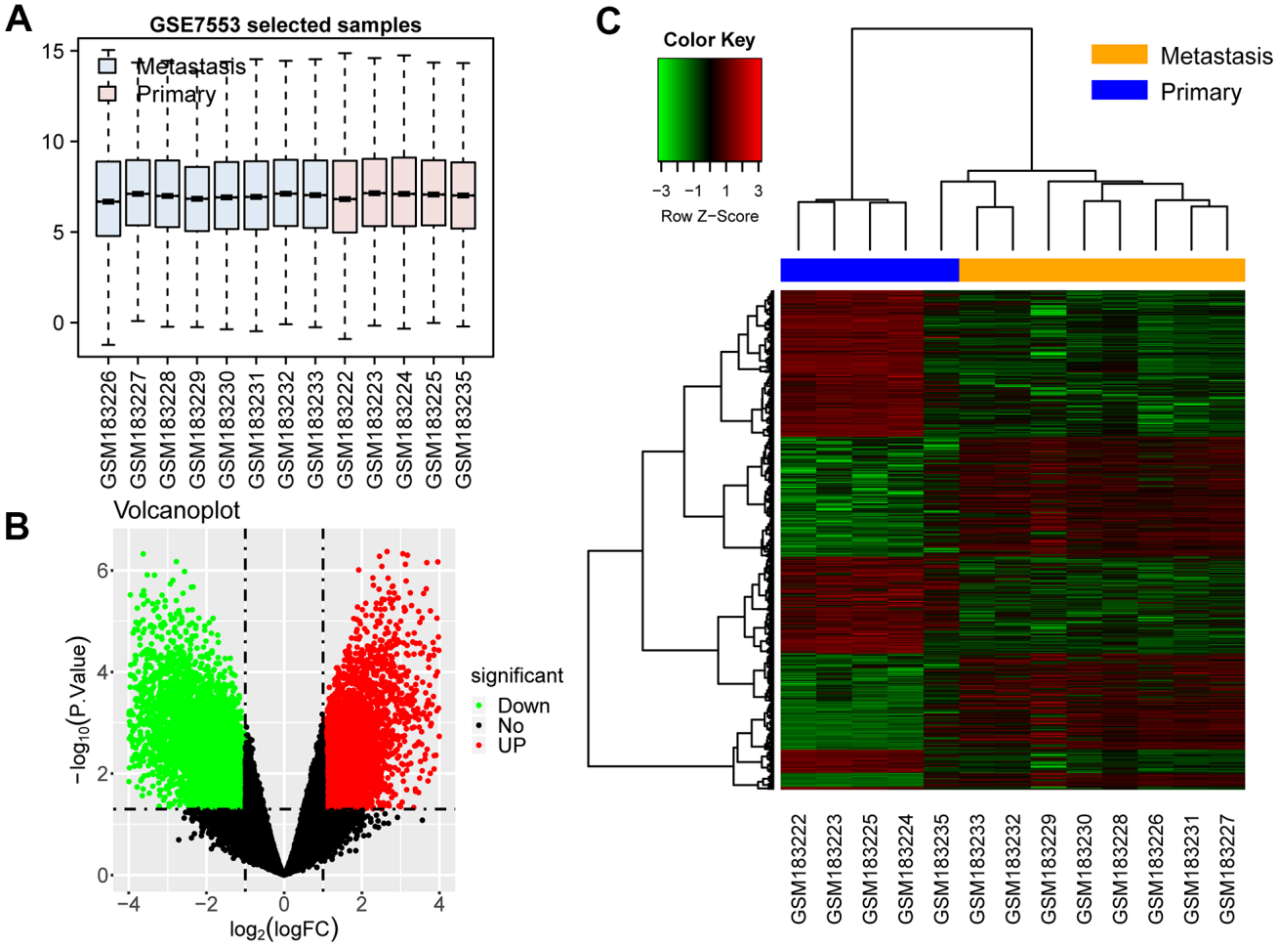
**DEGs in the two groups of samples.** (**A**) Box plot: Expression levels of all samples are normalized. (**B**) Volcano map of DEGs: the green dots represent downregulated genes in metastases, and the red ones represent upregulated genes. (**C**) Cluster heat map: the green blocks represent downregulated genes in metastases. The red blocks represent upregulated genes.

The expression data of the GSE7553 sequence were obtained from the GEO database, and the DEGs were calculated using Limma package. A total of 10,089 DEGs were screened out in accordance with the screening criteria. Among them, 4641 genes were upregulated (46.00%) and 5448 were downregulated (54.00%). The gplots software package was used to draw a box plot of sample expression. All DEGs were drawn using volcano maps and cluster heat maps (Figure 1). The results showed that the samples of melanoma metastases were clustered together. The samples of melanoma primary tumors were also clustered together, indicating that the samples in each group had similar expression patterns and high reproducibility.

### PI3K-AKT signaling pathway containing the largest number of DEGs

**Figure 2:**
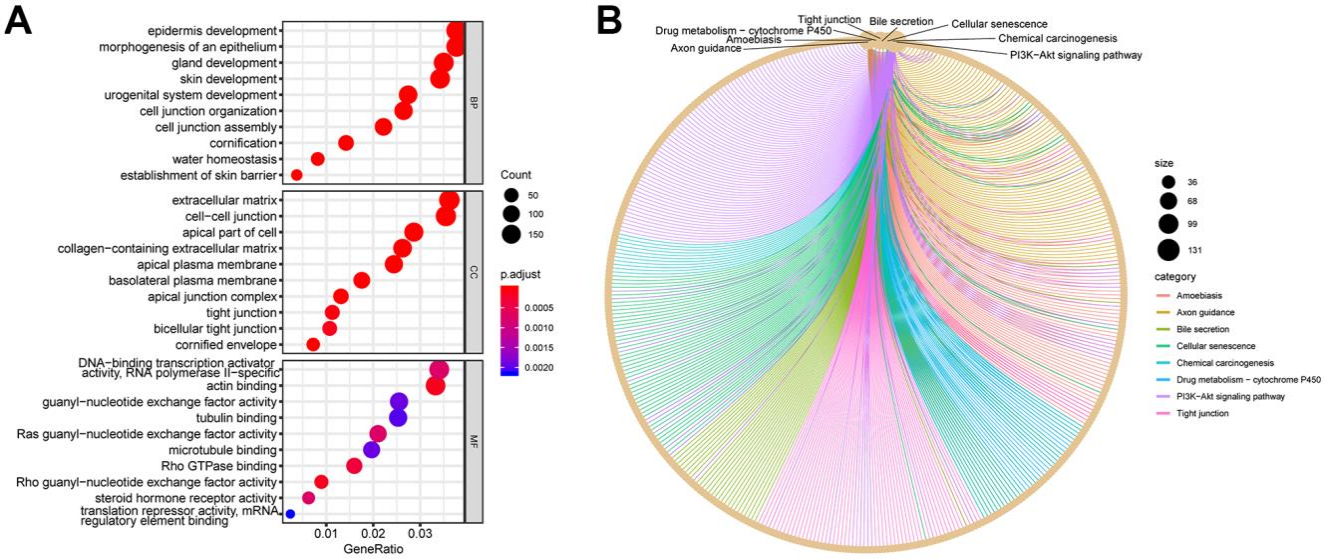
**GO and KEGG plots calculated using DEGs.** (**A**) GO dot plot: top 10 items in each category. (**B**) KEGG network plot: top eight pathway items calculated using DEGs. The surrounding dots represent genes.

A total of 10,089 DEGs were annotated via ClusterProfiler to further annotate the functions of DEGs and obtain the pathways that the DEGs participated in. This software package helped significantly enrich these DEGs into 1597 biological processes (BPs), including epidermal development, skin development, and epidermal cell differentiation; 166 cell components, including cell–cell junction, extracellular matrix, and cornified envelope; and 205 molecular functions, including Rho guanyl-nucleotide exchange factor activity, Ras guanyl-nucleotide exchange factor activity, and DNA-binding transcription activator activity (Figure 2A). ClusterProfiler was also used to enrich the Kyoto Encyclopedia of Genes and Genomes (KEGG) pathway for these DEGs, and the pathways important for this study were obtained. These 10,089 DEGs were significantly enriched in 74 KEGG pathways. Among them, the PI3K-AKT signaling pathway was enriched by the largest number of DEGs (131 DEGs). This pathway was also the focus of this study (Figure 2B). It contained the key genes *CDK2*, *CDK4*, *KIT*, and *Von Willebrand factor (VWF)*.

### Key genes leading to poor prognosis

**Figure 3:**
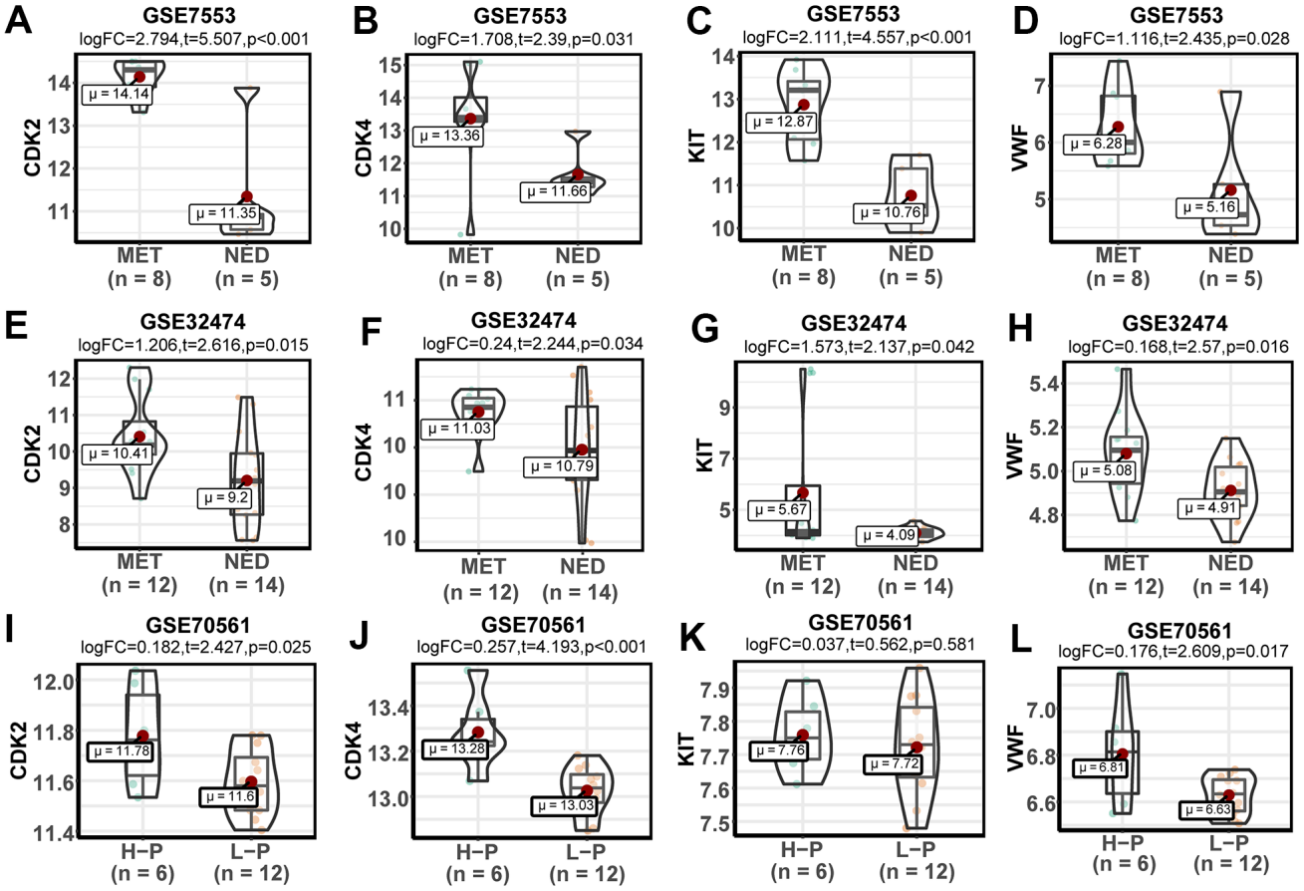
**Expression and verification of key genes.** (**A**–**D**) Violin plots of the expression levels of key genes in the GSE7553 dataset. (**E**–**H**) Violin plots of the expression levels of key genes in the GSE32474 dataset. (**I**–**L**) Violin plots of the expression levels of key genes in the GSE70561 dataset.

**Figure 4:**
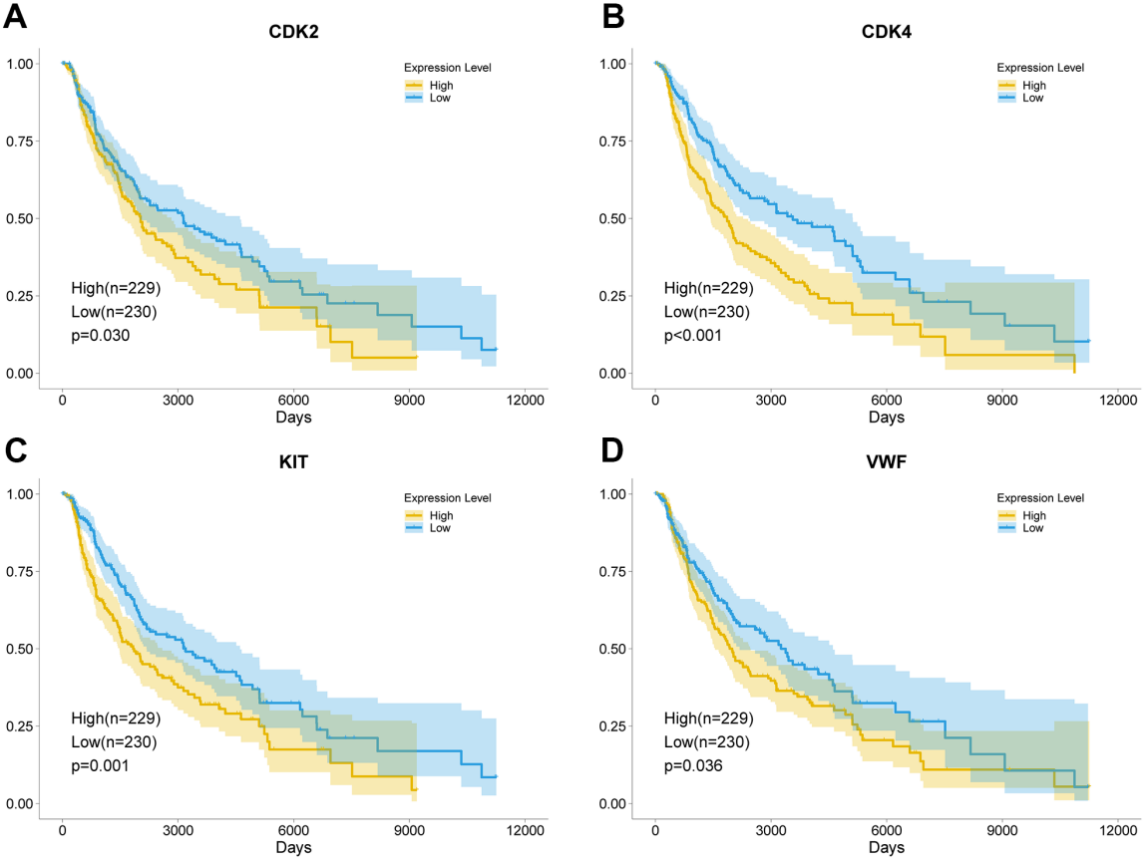
**Key genes leading to poor prognosis.** The high expression of *CDK2* (**A**), *CDK4* (**B**), *KIT* (**C**), and *VWF* (**D**) significantly reduced the survival rate of patients with melanoma. The yellow curve represents the high expression of the key genes, and the blue curve represents the low expression of the key genes.

**Figure 5:**
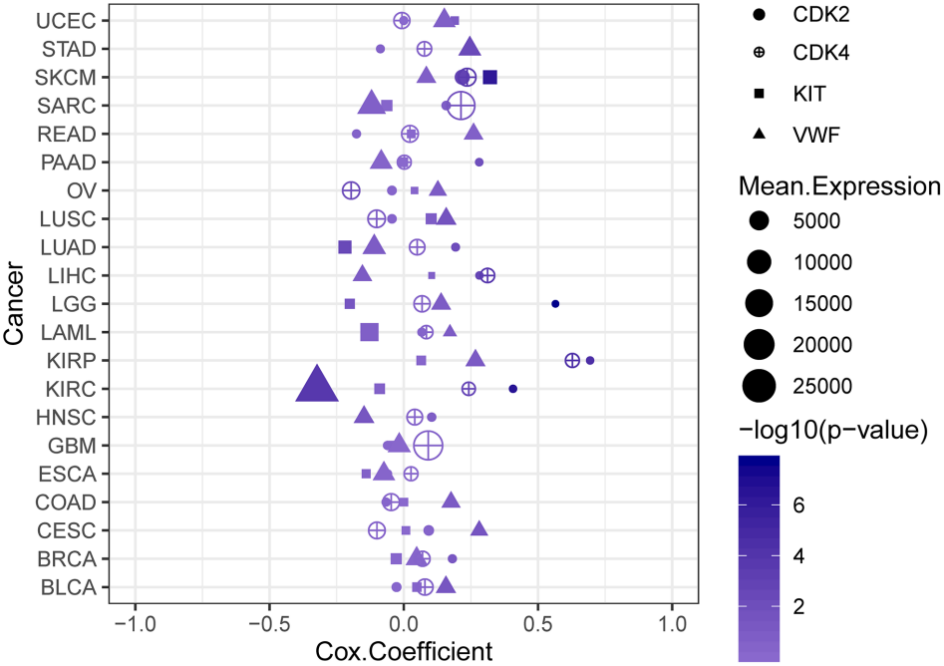
**Cox regression analysis plot.** The high expression levels of *CDK2*, *CDK4*, *KIT*, and *VWF* are risk factors for patients with melanoma.

Further analysis was conducted to verify the expression of DEGs and study the effect of the key genes on the prognosis of patients. In the GSE32474 dataset, the expression levels of *CDK2*, *CDK4*, *KIT*, and *VWF* in the melanoma metastasis group were significantly higher than those in the melanoma primary focus group (t = 2.616, 2.244, 2.137, and 2.570, respectively; logFC = 1.206, 0.240, 1.573, and 0.168, respectively; p < 0.05). The results were consistent with the analysis results in the GSE7553 dataset for *CDK2*, *CDK4*, *KIT*, and *VWF* (t = 5.507, 2.390, 4.557, and 2.435, respectively; logFC = 2.794, 1.707, 2.110, and 1.116, respectively; p < 0.05). Similarly, the expression levels of *CDK2*, *CDK4*, *KIT*, and *VWF* in the melanoma cells with high proliferation capabilities were higher than those in the melanoma cells with poor proliferation capabilities (t = 2.427, 4.193, 0.562, and 2.609, respectively; logFC = 0.182, 0.257, 0.037, and 0.176, respectively; p = 0.025, < 0.001, 0.581, and 0.017, respectively; Figure 3). The results of the survival analysis of patients with melanoma in TCGA demonstrated that the high expression of *CDK2*, *CDK4*, *KIT*, and *VWF* significantly reduced the survival rate of patients (Figure 4). Furthermore, according to the Cox regression analysis in the OncoLnc database, the Cox coefficients of *CDK2*, *CDK4*, *KIT*, and *VWF* were all > 0, indicating that the risk ratio > 1. Therefore, the high expression of the four key genes was a risk factor for patients with melanoma (Figure 5).

### Inactivation of *CDK4* or *VWF* inhibits the proliferation, migration, and invasion of melanoma

**Figure 6:**
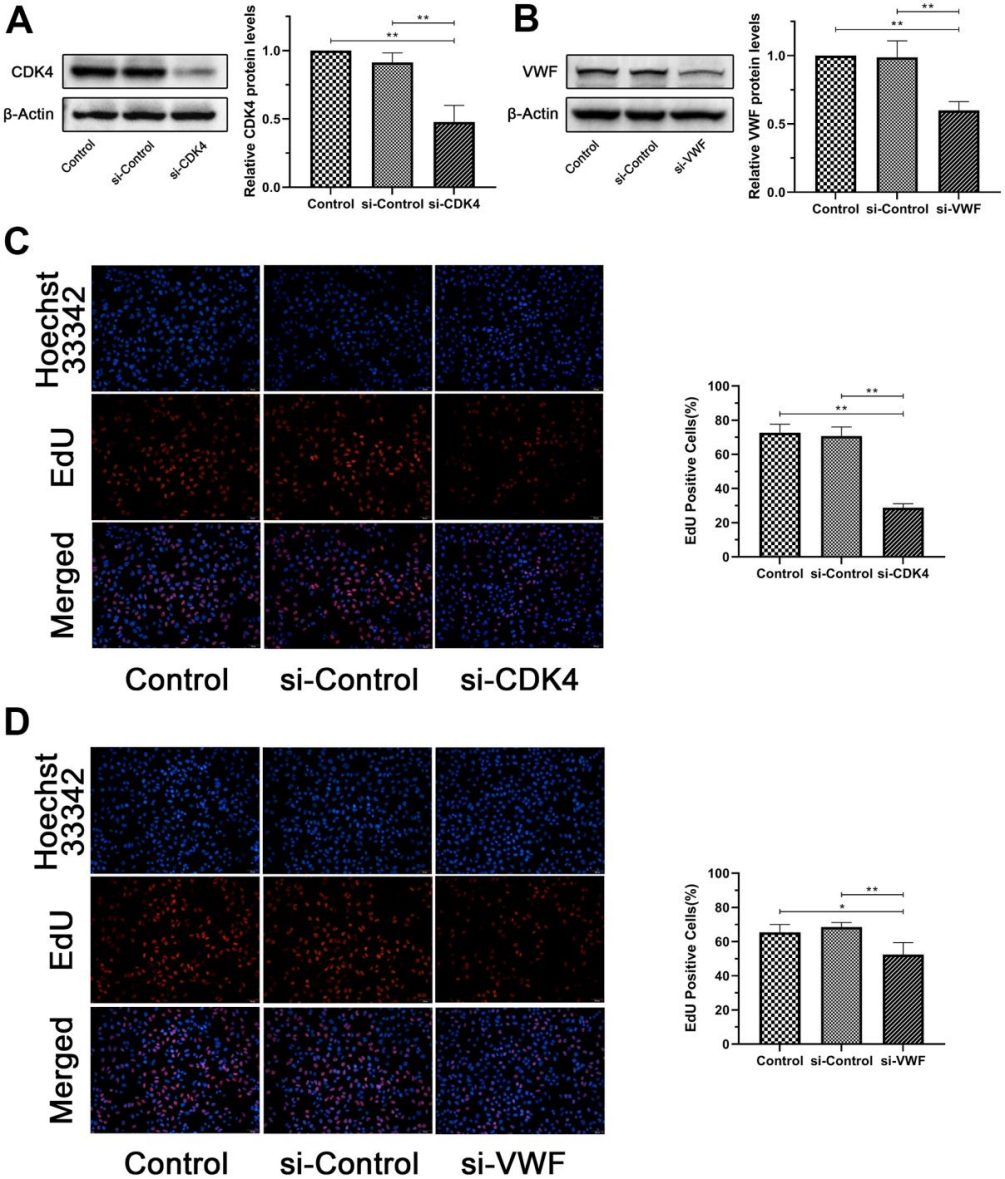
**Inactivation of *CDK4* or *VWF* inhibits the proliferation of melanoma.** (**A**, **B**) *CDK4* or *VWF* expression in the si-CDK4 or si-VWF group was significantly lower than that in the Control and si-Control groups. (**C**, **D**) EdU-positive cells of the si-CDK4 or si-VWF group was significantly reduced than that of the Control and si-Control groups. Asterisks indicate statistically significant difference (*: p<0.05, **: p<0.01, one-way ANOVA).

**Figure 7:**
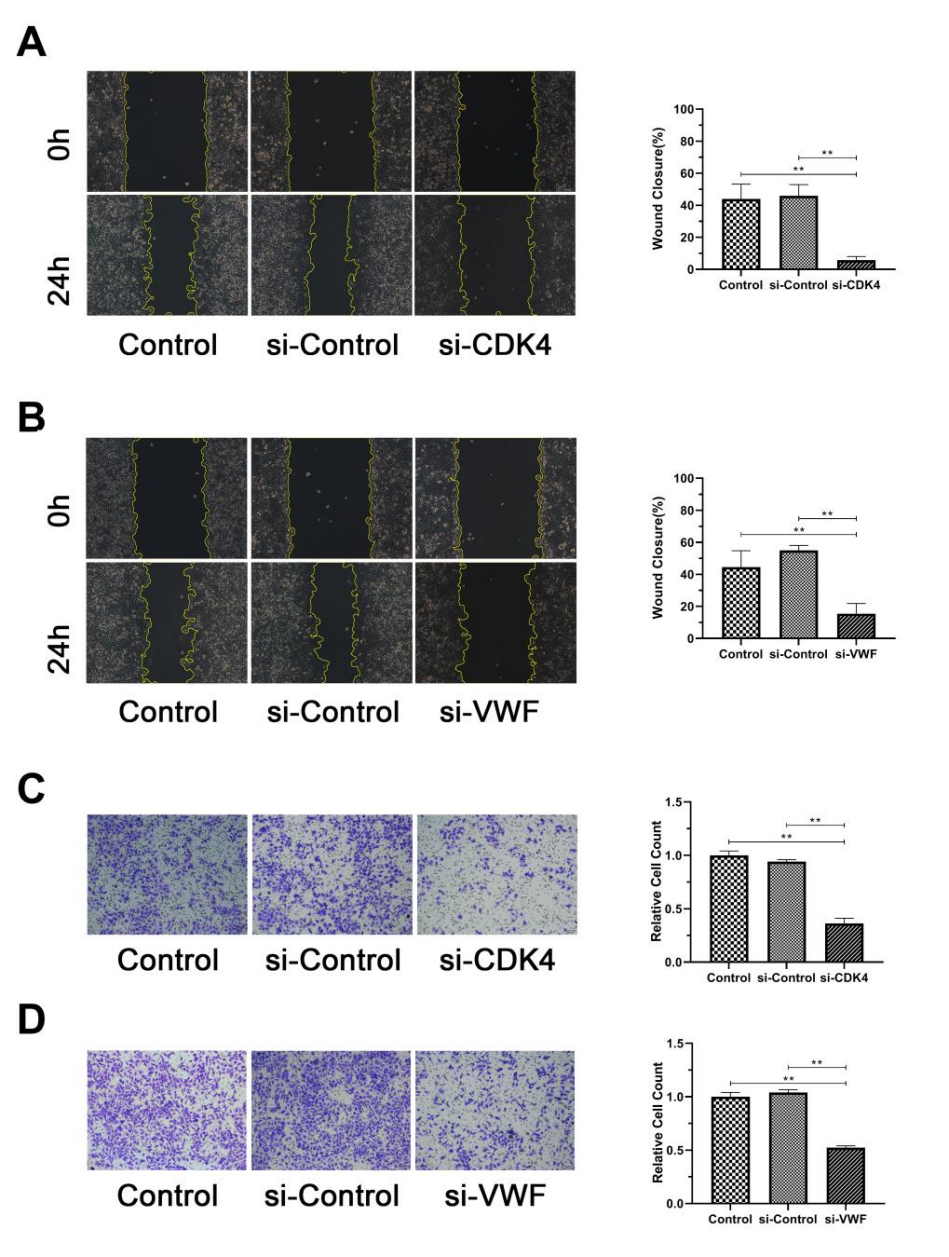
**Inactivation of *CDK4* or *VWF* inhibits the invasion and migration of melanoma.** (**A**, **B**) Closure percentage of the si-CDK4 or si-VWF group was significantly lower than that of the Control and si-Control groups. (**C**, **D**) Relative count of invading cells of the si-CDK4 or si-VWF group was significantly lower than that of the Control and si-Control groups. Asterisks indicate statistically significant difference (**: p<0.01, one-way ANOVA).

Representative results were selected to confirm the results of the above analysis for the functional verification of human melanoma cells. SiRNA silencing *CDK4* and *VWF* were transfected into MV3 cell line, and the silencing effect was verified by Western blot analysis. The results showed significantly lower *CDK4* or *VWF* expression in the si-CDK4 or si-VWF group, respectively, than in the Control and si-Control group (Figure 6A, 6B). EdU, transwell, and wound healing assays were conducted to verify whether *CDK4* or *VWF* inactivation can affect the proliferation, migration, and invasion of melanoma cells. The results from EdU assay showed that the number of EdU-positive cells was significantly reduced in the si-CDK4 or si-VWF group than in the Control and si-Control groups (Figure 6C, 6D). The findings from wound healing and transwell assays revealed that the closure percentage and the number of invading cells were significantly lower in the si-CDK4 or si-VWF group than in the Control and si-Control groups (Figure 7A–7D). All these results showed that *CDK4* or *VWF* inactivation inhibits the proliferation, migration, and invasion of melanoma.

### Regulation of key genes

**Figure 8:**
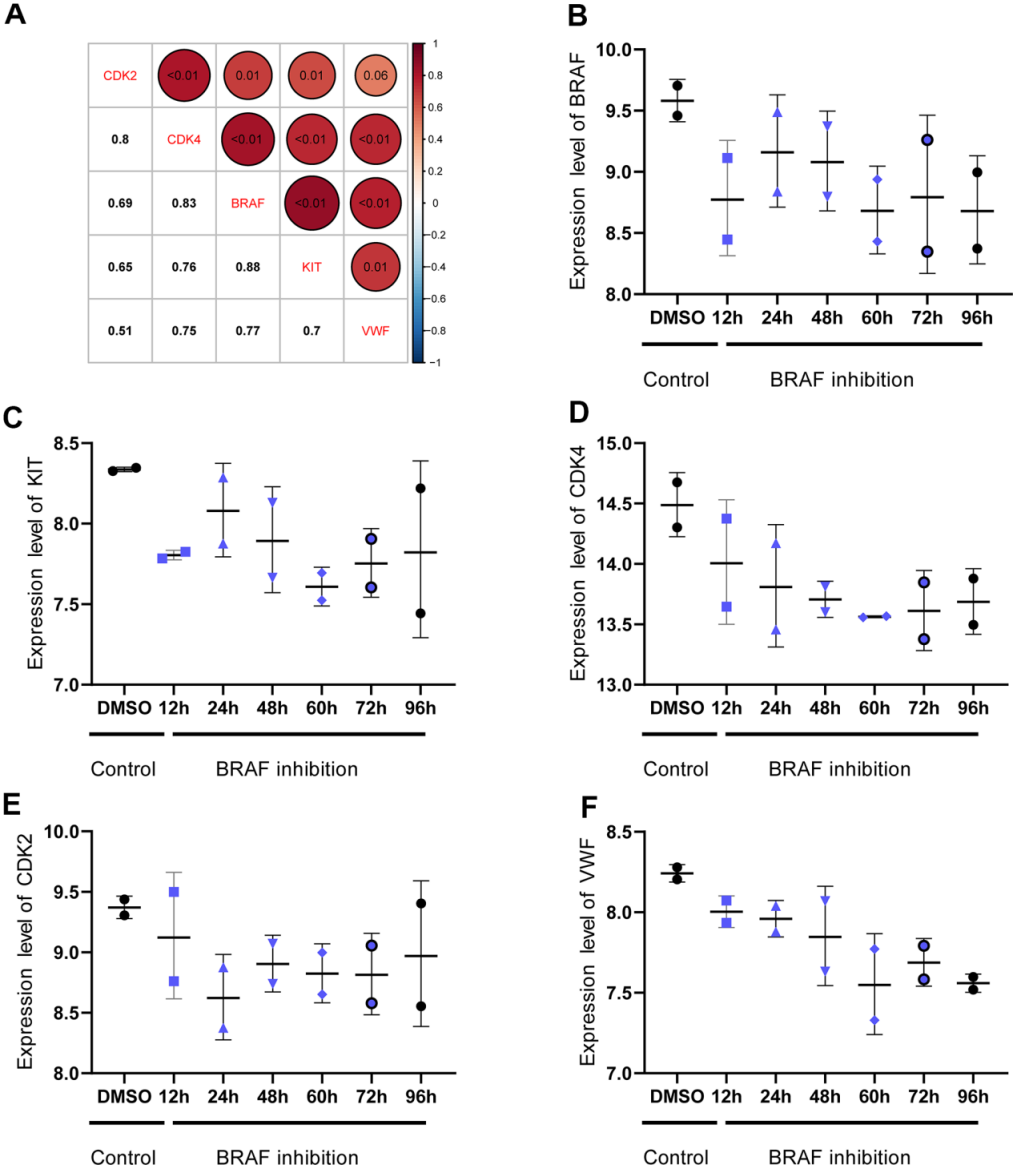
**Correlation between *BRAF^V600E^* expression and expression of key genes.** (**A**) The expression of *CDK2*, *CDK4*, *KIT*, and *VWF* is significantly correlated with that of *BRAF^V600E^*. The numbers in the upper right corner represent the p value, and the numbers in the lower left corner represent the correlation coefficient. (**B**–**F**) Expression trend of *CDK2*, *CDK4*, *KIT*, and *VWF* after inhibiting the expression of *BRAF^V600E^*.

**Figure 9:**
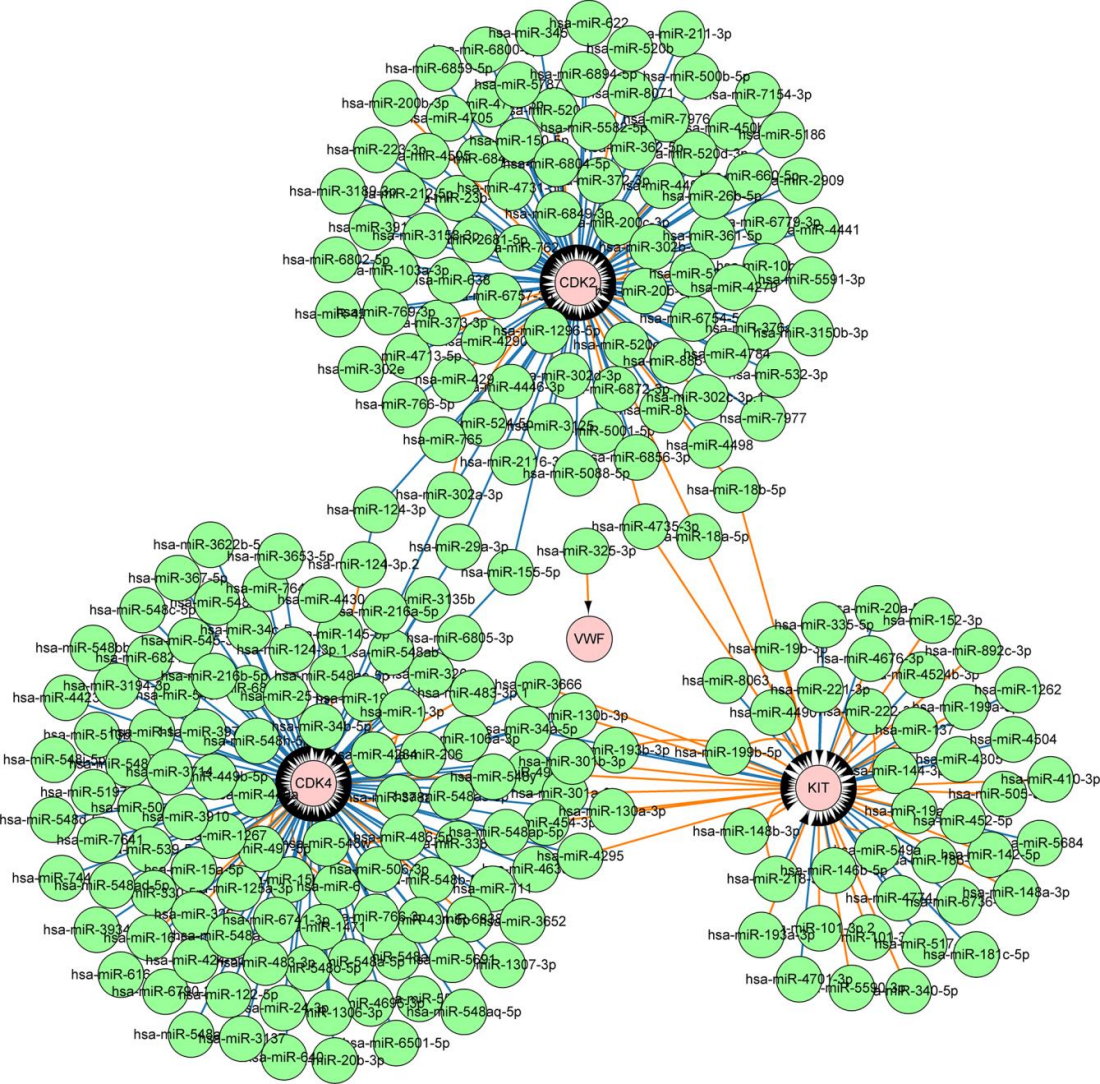
**MiRNAs involved in the regulation of four key genes.** A total of 240 miRNAs may regulate the expression of four key genes.

Correlation analysis of gene expression was performed to analyze the microRNA (miRNA) regulatory network of the key genes and further analyze their regulation. First, the GSE58721 microarray data containing samples treated with *BRAF^V600E^* inhibitor PLX4720 were analyzed. The results showed that the expression levels of *CDK2*, *CDK4*, *KIT*, and *VWF* showed a trend of decreasing and then gradually becoming stable compared with those in the control group. This finding indicated that the expression of the four key genes was closely related to that of *BRAF^V600E^* (Figure 8). Next, the CyTargetLinker application in Cytoscape, using the miRTarBase 8.0 and TargetScan 7.2 databases, was utilized to draw a visual view of the potential relationship between miRNA and DEGs. The results showed 240 miRNAs that may regulate the expression of the key genes (Figure 9).

## DISCUSSION

The incidence of melanoma among young and middle-aged individuals is higher than that of other solid tumors, but the overall incidence of melanoma screened for age and population climbs steadily and peaks at the seventh and eighth decades of life [18, 19]. The global incidence of skin melanoma has been recently increasing at a faster rate every year compared with that of other cancers [20]. In addition, melanoma often develops brain metastases, second only to lung cancer and breast cancer. Brain metastases occur in up to 75% of patients with melanoma, and the mortality rate of brain metastases is 95% [21]. The occurrence of tumor is the result of multiple genes participating together, and the regulation at the transcriptional level plays a key role [22]. Current studies have shown that the metastasis of melanoma is related to the expression of certain genes and the activation of certain pathways, such as ABCB5 expression, lncRNA KCNQ1OT1, the MMP-9 signaling pathway, and the HGF-MET signaling pathway [23–26]. However, the potential relationship between melanoma metastasis and gene expression is still not fully understood.

In this study, the GSE7553 dataset was used to calculate DEGs, and Gene Ontology (GO) and KEGG enrichment analyses were performed on the DEGs between melanoma metastases and primary tumors. The results showed that the PI3K-Akt signaling pathway contained the largest number of DEGs. This pathway was also involved in the process of melanoma metastasis. After screening methods, such as gene expression level verification, survival analysis, and regulatory analysis, were combined, four key genes were finally obtained.

The expression levels of *CDK2*, *CDK4*, *KIT* and *VWF* were verified through the analysis of the GSE32474 dataset, and the results were consistent with those in the GSE7553 dataset. The four key genes were significantly upregulated in the metastasis samples. Similarly, in the 501Mel cell line, which is a melanoma cell line with proliferation phenotype, the expression levels of these key genes were also upregulated. Analysis of the clinical data of patients with melanoma in TCGA database showed that the upregulation of the key genes reduced the OS rate of patients with melanoma. Cell and molecular biology experiments, such as 5-ethynyl-20-deoxyuridine, wound healing, and transwell assays, were also conducted to verify the representative results of the above analysis. The inactivation of key genes was found to inhibit the proliferation, migration, and invasion of melanoma. These results indicated that the four key genes may play an important role in the occurrence, development, and metastasis of melanoma, and they could be used as prognostic markers.

The PI3K-Akt signaling pathway is one of the pathways that cause almost all human cancers. New drugs targeting this pathway may further improve the current research results through more effective selectivity and efficacy [27]. The activation of the PI3K-Akt signaling pathway enhances cancer cell proliferation, which is accompanied by increased expression of cell cycle regulatory factors [28]. The three types of cell cycle regulators are cyclins, cyclin-dependent kinases (CDKs), and cyclin-dependent kinase inhibitors [29]. CDKs are at the center of the steps of cell cycle regulation, and they play an important role in cell proliferation [30]. The role of CDK2 and CDK4 is to promote the transition from G1 phase to S phase during cell proliferation. Cyclin D is activated first and then combined with CDK4 or CDK6 to activate CDK4 or CDK6. This step works in the early G1 phase [31]. These two factors induce the expression of cyclin E and *CDK2* to form a cyclin E-CDK2 complex, which functions in the middle and late G1 phase, and promotes the cell proliferation process to enter the S phase and initiates DNA replication [32]. Studies have shown that the overexpression of CDKs could cause the tumor proliferation to go out of control and lead to tumor malignancy and metastasis [33, 34]. Inhibiting or downregulating the expression of *CDK2* and *CDK4* delays or inhibits cell cycle progression and reduces tumor proliferation and metastasis [35–37]. This finding was also consistent with the results of the present research. By grouping the *CDK2* and *CDK4* expression levels of melanoma samples, survival analysis of patients in the high- and low-expression groups showed that the high expression of *CDK2* and *CDK4* significantly reduced the OS rate of patients with melanoma.

In the enrichment results of the PI3K-Akt signaling pathway, the highly expressed *KIT* and *VWF* also attracted the attention of the researchers. KIT is a type III receptor tyrosine kinase than could interact with various proteins and mediate the activation of the PI3K-Akt signaling pathway. It plays a vital role in the development of tumors [38]. The increased expression of *KIT* enhances the proliferation and metastasis of breast cancer, rectal cancer, and other cancer cells [39, 40]. Inhibiting the expression of *KIT* could improve the prognosis of patients with cancer [41, 42]. Studies have shown that in patients with melanoma, *KIT* variants could be classified as a rare subtype [43, 44]. *VWF* is also important in the present research. It is a complex multimeric plasma glycoprotein that mediates platelet adhesion and thrombus formation and plays an important role in the coagulation process [45]. Increasing evidence showed that the increased expression of *VWF* not only could lead to an increase in the incidence of cancer complications in terms of thrombosis but also interact with various cancer cells, such as those in papillary thyroid carcinoma, osteosarcoma, and gastric cancer. It could also promote cancer cell proliferation, leading to cancer progression and metastasis [46–48]. Some clinical studies have found that the increased expression of *VWF* often indicates poor prognosis for patients with cancer [49, 50]. Cancer cell metastasis could be reduced by inhibiting cancer cell-derived VWF in tumor cells [51, 52]. In the present study, the expression of *KIT* and *VWF* in the melanoma metastasis samples significantly increased. Survival analysis showed that the OS rate of patients with melanoma in the high expression group of *KIT* and *VWF* was significantly reduced. Furthermore, according to the result of Cox regression analysis, the high expression of the four key genes was a risk factor for patients with melanoma. Studies have shown that patients with melanoma have an increased risk of kidney cancer, and a high risk of melanoma exists among patients with renal cell carcinoma. These studies proved a bidirectional association between RCC and MM [53, 54]. In the present study, apart from SKCM, kidney renal papillary cell carcinoma exhibited CDK2, CDK4, KIT, and VWF Cox coefficient values of > 0, indicating that the high expression of these four key genes is a risk factor for patients with renal carcinoma. This finding is also consistent with the results of the above studies.

BRAF is a component of the MAPK signaling pathway; it could activate the downstream MEK1 and MEK2 and further lead to the proliferation and metastasis of melanoma [55]. However, developing drug resistance to *BRAF^V600E^* inhibitors is common in patients with melanoma [56]. The results of correlation analysis in the present study showed that the inhibition of *BRAF^V600E^* could significantly lead to the downregulation of the four key genes. The expression pattern of *BRAF^V600E^* was closely related to these four key genes, indicating that the expression of the latter may be partially regulated by *BRAF^V600E^*. In melanoma cells with or without *BRAF^V600E^* inhibitor resistance, inhibiting the expression of *CDK2*, *CDK4*, *KIT*, and *VWF* may become a new choice to inhibit the progression of melanoma. MiRNA is small molecule non-encoded RNA. It regulates gene expression post-transcriptionally and suppresses targeted mRNA expression [57]. Studies have shown that miRNA w involved in the regulation of numerous BPs. In the present study, a total of 240 miRNAs were involved in the regulation of the four key genes. Studies have shown that these miRNAs could target the expression of key genes to inhibit the growth of cancer cells. For example, miR-124-3p reduced the progress of hepatocellular carcinoma by inhibiting the expression of target genes, such as *CDK2* and *CDK4* [58]. By targeting *CDK2*, miR-885-5p also induced neuroblastoma cell apoptosis and aging [59]. In the study of gastrointestinal stromal tumors, miR-124-3p reduced the viability of tumor cells by inhibiting *KIT* expression and induced tumor cell apoptosis [60]. Regulating the expression of these miRNAs may also affect the proliferation and metastatic ability of melanoma cells.

The results of this study showed that the PI3K-Akt signaling pathway and the key genes *CDK2*, *CDK4*, *KIT*, and *VWF* promoted the proliferation and metastasis of melanoma, and they were significantly related to the prognosis of patients with melanoma. However, further biological and clinical experiments are needed to confirm these results.

## MATERIALS AND METHODS

### Microarray data and calculation of DEGs

Microarray data from the GSE7553 sequence (GPL570, Affymetrix Human Genome U133 Plus 2.0 Array) were obtained, and the first group containing melanoma was used. This group contained eight samples of melanoma metastases and five samples of melanoma primary lesions. The gene expression sequence was imported into R Studio, and the Limma (version 3.38.3) software package was used to calculate the DEGs [fold change (FC) > 2.0 or < −0.5 and p < 0.05 as screening criteria] [61]. The gplots software package (version 3.0.1.1) was used to draw box plots, volcano plots, and cluster heat maps on the basis of DEG expression [62].

### Functional annotation of DEGs

ClusterProfiler, a software package based on R language, was used for GO annotation and KEGG pathway enrichment analysis of DEGs [63], and the enrichplot (version 1.2.0) software package was used to visualize the calculated enrichment analysis results.

### Verification and survival analysis

Samples of 12 melanoma metastases and 14 melanoma primary lesions in the GSE32474 sequence were used for analysis to verify the expression levels of the key genes, and the results were compared with the expression levels obtained via GSE7553 analysis. In the GSE70561 sequence, the 501 MEL cell line used for sequencing was a recognized proliferative phenotype of melanoma cell line. The six samples in the control group consisted of cells with high proliferation capabilities, and the 12 samples in the experimental group were cells with poor proliferation capabilities. The sequencing data of the samples in the two groups were used to analyze the relationship between gene expression levels and proliferation capabilities. In addition, the expression of the key genes in all melanoma samples in TCGA database was divided in accordance with the median, and survival analysis was performed on the two groups of patients. The Cox regression analysis results of melanoma and other common tumors were further obtained from the OncoLnc database (http://www.oncolnc.org/download/) derived from TCGA, and the results were plotted using the ggplot2 software package [64].

### Cell transfection

Human melanoma cell line MV3 was purchased from the Chinese Academy of Sciences Stem Cell Bank (Shanghai, China). MV3 cells were seeded in a six-well plate (5 × 10^5^ cells per well) and then placed in a 37° C incubator overnight. *CDK4* and *VWF* expression was regulated by using sequence-specific siRNA and siRNA-mate transfection reagents (Gene Pharma, Shanghai, China) (siRNA sequence targeting *CDK4*: 5ʹ-CUCUUAUCUACAUAAGGAUTT-3ʹ; siRNA sequence targeting *VWF*: 5ʹ-GGCUUGCACCAUUCAGCUATT-3ʹ). The cells were classified into following groups: Control, si-Control with empty vector, si-CDK4 with *CDK4* interference vector, or si-VWF with *VWF* interference vector.

### Western blot analysis

Procedures involving RIPA buffer and protease inhibitors were used to extract and denature proteins from cells. Protein concentration was detected using a BCA reagent. After electrophoresis and membrane transfer operations, the PVDF membrane was sealed in a fast blocking solution for 30 minutes at room temperature, then incubated with CDK4 antibody, VWF antibody, and β-actin antibody (Proteintech, Chicago, IL, USA) on a shaker overnight at 4° C, and finally washed with TBST for three times for 5 minutes each. Finally, the PVDF membrane and the secondary antibody (Proteintech, Chicago, IL, USA) were incubated for 1 hour at room temperature, and an enhanced chemiluminescence (ECL) kit was employed for visualization.

### 5-ethynyl-20-deoxyuridine assay

The same number of cells from the three groups were incubated with 5-ethynyl-20-deoxyuridine (EdU) for 2 hours in accordance with the manufacturer's instructions. After being washed once with PBS, the cells were treated with 100 μL of 1X Apollo reaction mixture for 30 minutes. DNA was stained with 100 μL of 1X Hoechst 33342 for 30 minutes and observed under a fluorescence microscope.

### Wound healing assay

MV3 cells were seeded in a six-well plate, transfected, and processed. After 24 hours, a straight line was drawn with a sterile 200 μL pipette tip, and 10% FBS medium was added. Images of scratched cells were captured using a microscope at 0 and 24 hours later.

### Transwell assay

After 24 hours of designated treatment, the digested cells were resuspended in serum-free medium. Afterward, 100 μL of cell suspension (2 x 10^5^) was added to the upper chamber of the Transwell culture plate, and 600 μL of medium containing 10% FBS was added to the bottom chamber. The 24-well plate was incubated at 37° C and 5% CO2 for 24 hours. The cells on the upper surface of the membrane were gently wiped off with a wet cotton swab. The upper chamber was carefully taken out and placed in 4% paraformaldehyde to allow the cells to be fixed for 10 minutes. After being stained with crystal violet for 10 minutes, the number of cells was counted under an optical microscope.

### Analysis of regulatory mechanisms

The GSE58721 microarray data contained samples that inhibited *BRAF^V600E^* expression. These data were used to analyze the effect of inhibiting *BRAF^V600E^* expression on the expression of key genes obtained from the above analysis. Corrplot software package was used to analyze the correlation between *BRAF^V600E^* and the key genes [65]. The CyTargetLinker application (including miRTarBase 8.0, TargetScan 7.2) in Cytoscape was used to obtain the miRNAs that regulate the key genes [66].

### Statistical analysis

R language was used for statistical analysis. DEGs were screened out using Bayes test. A risk ratio model was utilized for survival analysis, and a Kaplan–Meier curve was further drawn. Pearson correlation test was used for correlation analysis. One-way ANOVA was used to compare multiple groups. A p value of less than 0.05 was considered statistically significant.

### Data availability statement

The datasets supporting the conclusions of this article are available in the GEO repository (http://www.ncbi.nlm.nih.gov/geo/) and TCGA database (https://portal.gdc.cancer.gov/).
